# A well supported multi gene phylogeny of 52 dictyostelia

**DOI:** 10.1016/j.ympev.2019.01.017

**Published:** 2019-05

**Authors:** Christina Schilde, Hajara M. Lawal, Koryu Kin, Ikumi Shibano-Hayakawa, Kei Inouye, Pauline Schaap

**Affiliations:** a School of Life Sciences, University of Dundee, Dundee DD15EH, UK; b Department of Physics, Graduate School of Science, Kyoto University, Kyoto 606-8502, Japan; c Department of Botany, Graduate School of Science, Kyoto University, Kyoto 606-8502, Japan

**Keywords:** Phylogenomics, Ancestral state reconstruction, Phylogenetic marker genes, Dictyostelia, *Polysphondylium multicystogenum*, *Dictyostelium caveatum*

## Abstract

The Dictyostelid social amoebas are a popular model system for cell- and developmental biology and for evolution of sociality. Small subunit (SSU) ribosomal DNA-based phylogenies subdivide the known 150 species into four major and some minor groups, but lack resolution within groups, particularly group 4, and, as shown by genome-based phylogenies of 11 species, showed errors in the position of the root and nodes separating major clades. We are interested in the evolution of cell-type specialization, which particularly expanded in group 4. To construct a more robust phylogeny, we first included 7 recently sequenced genomes in the genome-based phylogeny of 47 functionally divergent proteins and next selected 6 proteins (Agl, AmdA, PurD, PurL, RpaA, SmdA) that independently or in sets of two fully reproduced the core-phylogeny. We amplified their coding regions from 34 *Dictyostelium* species and combined their concatenated sequences with those identified in the 18 genomes to generate a fully resolved phylogeny. The new AAPPRS based phylogeny (after the acronym of the 6 proteins) subdivides group 4 into 2 branches. These branches further resolve into 5 clades, rather than the progressively nested group 4 topology of the SSU rDNA tree, and also re-orders taxa in the other major groups. Ancestral state reconstruction of 25 phenotypic traits returned higher “goodness of fit” metrics for evolution of 19 of those traits over the AAPPRS tree, than over the SSU rDNA tree. The novel tree provides a solid framework for studying the evolution of cell-type specialization, signalling and other cellular processes in particularly group 4, which contains the model Dictyostelid *D. discoideum.*

## Highlights

• A *Dictyostelium* phylogeny inferred from 18 genomes resolves positions of all major and minor groups.
• A tree based on amplified sequences for 6 proteins over 34 species subdivides group 4 into five clades.
• Scenarios for trait evolution are more likely over the 6-protein tree than over an SSU rDNA tree.

## Graphical abstract

**Figure:**
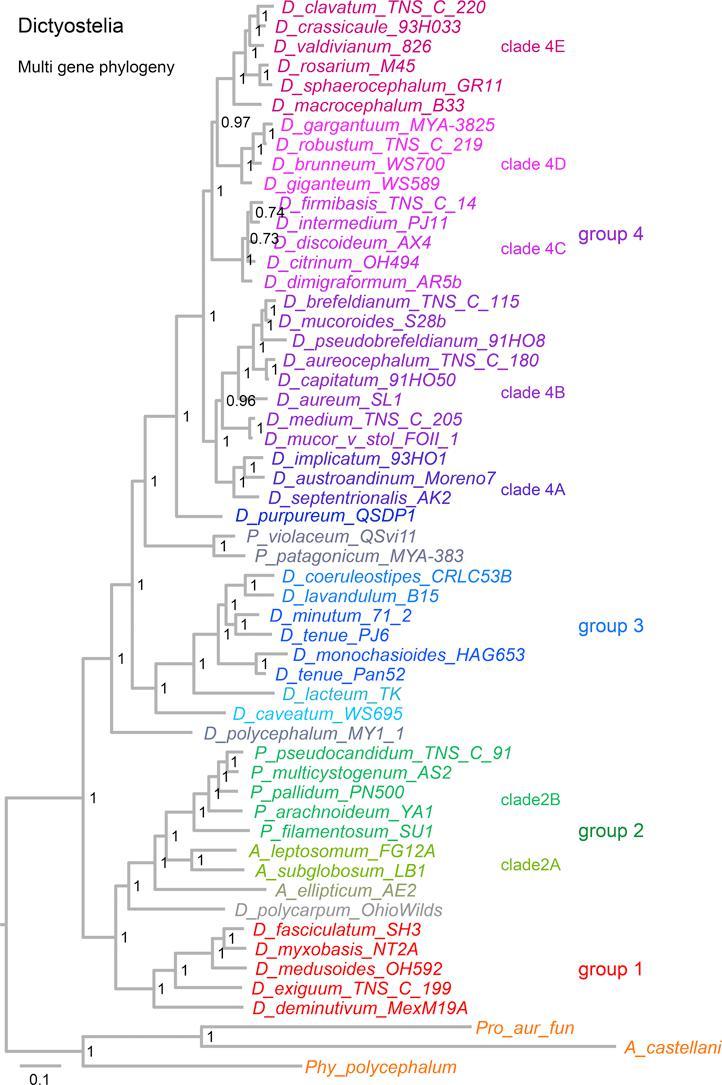

## 1 Introduction

To investigate how any biological process evolved, it is essential to understand the phylogenetic relationships of the group of organisms under study. We are interested in the evolution of early multicellularity and cell-type specialization, using the dictyostelid social amoebas as a genetic model system. Dictyostelia are unicellular while feeding, but aggregate to form multicellular fruiting structures when their bacterial food source is depleted. Inside the emerging fruiting structures, the amoebas differentiate into dormant spores and vacuolated stalk cells, which are contained within a cellulose tube, and hold the spore mass aloft. Within Dictyostelia, one clade forms an empty cellulose tube without vacuolated stalk cells, while within taxon group 4, which contains the model *Dictyostelium discoideum*, three additional cell types emerged, which respectively form a basal disc to support the stalk and an upper and lower cup that anchors the spore mass to the stalk. These innovations accompanied the general trend in this group towards larger and more robust fruiting structures (Romeralo et al., 2013).

The first molecular phylogeny inferred from SSU rDNA and α-tubulin sequences subdivided the then ∼100 known species of Dictyostelia into 4 major groups, but left the position of the root and of some group-intermediate species unresolved (Schaap et al., 2006). Elaboration of the SSU rDNA phylogeny with 50 novel species indicated that the group-intermediate species might actually represent minor groups (Romeralo et al., 2011). The sequencing of genomes representative of the four major groups (Eichinger et al., 2005, Gloeckner et al., 2016, Heidel et al., 2011, Sucgang et al., 2011) enabled the inference of a core phylogeny from 47 concatenated functionally divergent genes, which robustly placed the root of Dictyostelia between two major branches that contained groups 1 and 2 and groups 3 and 4, respectively (Romeralo et al., 2013), which was confirmed by a 213 gene phylogeny (Sheikh et al., 2015). A hybrid tree of the latter phylogeny of five taxa and a SSU rDNA tree of 191 species and strains was used as the basis for a novel classification of Dictyostelia in which groups 1, 2, 3 and 4, inclusive of the minor “violaceum complex”, were ranked as families and named Cavenderiaceae, Acytosteliaceae, Raperosteliaceae and Dictyosteliaceae, respectively (Sheikh et al., 2018), but leaving the positions of several minor groups, now assigned the rank of genus, unresolved.

Custom sequencing of five more genomes of group-intermediate or clade-intermediate species resulted in a robust core phylogeny with the intermediate “violaceum complex” as the sister to group 4, the “polycephalum complex” as sister to group 3 and the “polycarpum complex” as sister to group 2 (Singh et al., 2016). While this phylogeny was constructed from the same 47 genes as before (Romeralo et al., 2013), further analysis showed that inference from five sets with as little as 10 randomly selected genes already replicated the 47 gene phylogeny with full support. While SSU rDNA on its own was relatively unsuccessful in reproducing the consensus 47 core gene phylogeny, two single genes and four sets of two concatenated genes, which individually yielded trees with a single non-consensual node, each replicated the consensus phylogeny. This indicated that smaller gene sets can be used for reliable phylogenetic inference (Singh et al., 2016).

We are particularly interested in events that occurred within group 4 to cause the appearance of three novel somatic cell types. Group 4 also differs from the “violaceum” complex and all other groups in the pre-patterning of its future spore and stalk cells during the migrating “slug” stage, the use cAMP as chemoattractant for aggregation and the loss of encystation as alternative survival strategy (Romeralo et al., 2013, Schilde et al., 2014). The relationship between species within group 4 and its interface with the “violaceum” complex can currently only be inferred from the poorly resolved SSU rDNA phylogeny. To increase the reliability of this part of the phylogeny in a cost-effective manner, we have amplified regions of 6 genes, which were validated in the earlier work (Singh et al., 2016), to infer a phylogeny from 27 species in group 4 and the “violaceum complex”. The new phylogeny resolves almost all nodes within group 4 with 100% statistical support, but deviates considerably from the SSU rDNA based phylogeny. We have also amplified and included the same genes from a smaller set of species within the other major and minor groups in the tree, indicating that also here clades should be re-ordered, but reproducing the topology of the 47-gene core phylogeny. The novel tree offers researchers a reliable framework for studying the evolution of developmental or cellular processes.

## 2 Material and methods

### 2.1 Cell culture and DNA extraction

Most species used in this work (See appendix A, Table A1) were sent to us by the field biologists Drs. H. Hagiwara and J. C. Cavender for construction of the first SSU rDNA phylogeny (Schaap et al., 2006) and were revived from frozen stocks. Species were grown in association with *Klebsiella aerogenes* on 1/5th SM agar (2 g BACTO™ peptone (DIFCO), 0.2 g yeast extract, 2 g glucose, 0.2 g MgSO_4_·7H_2_O, 2.2 g KH_2_PO_4_, 1.25 g Na_2_HPO_4_·2H_2_O and 15 g agar per litre H_2_O). For use of DNA in gene amplification by PCR, cells were harvested from growth plates and washed twice with 10 mM Na/K phosphate buffer, pH 6.5 (PB). DNA was extracted from approximately 1-2 × 10^6^ cells using the GenElute Mammalian genomic DNA extraction kit (Sigma-Aldrich).

### 2.2 PacBio sequencing and assembly of the *P. multicystogenum* genome

*P. multicystogenum* AS2 (Kawakami and Hagiwara, 2008) was grown as described above, but after growth cells were plated on non-nutrient agar and additionally starved overnight at 4 °C and 2 h at 21 °C to clear remaining bacteria. Genomic DNA was isolated from purified nuclei as described previously (Gloeckner et al., 2016). A PacBio 20/30 kilobase (kb) genomic DNA library was prepared from *P. multicystogenum* genomic DNA and sequenced using the PacBio RS II sequencing platform by the Earlham Institute, Norwich, UK. Sequencing was performed with C4-P6 chemistry on 1 SMRT cell yielding 15x coverage of the 30 megabase genome. Data quality control, basecalling, and formatting as well as HGAP data quality control were performed at the Earlham Institute. Raw reads were assembled using Canu (Koren et al., 2017) with a parameter setting recommended for low coverage (<20x) data. Specifically, the corrected error rate of reads was set to be 0.075, with other parameters set as default. The final assembly consisted of 596 contigs, totalling about 30 megabases, with an N50 of 81.6 kilobases, and is available from Genbank as bioproject PRJNA495730.

### 2.3 Illumina sequencing of the *D. caveatum* genome

*D. caveatum* WS-695 (B4-3) (Waddell, 1982) was grown on SM agar with *K. aerogenes*, extensively washed to remove bacteria, and starved for ∼3 h. Genomic DNA was prepared using GenEluteTM Genome DNA Miniprep Kit (Sigma-Aldrich, St. Louis, MO, USA), followed by RNAse treatment. The integrity of DNA was checked by agarose gel electrophoresis. Genome sequencing was conducted on the Illumina HiSeq platform by the paired-end method with 100-bp reading (Hokkaido System Science, Sapporo, Japan). After removal of adapter sequences with Skewer v.0.1.123, the reads aligned to the *K. aerogenes* genome (Shin et al., 2012) were removed using bwa-0.7.12, and the remaining reads were assembled using Velvet-1.2.10 (Zerbino and Birney, 2008, Zerbino et al., 2009). The final assembly consisted of 2180 contigs, with an N50 of 40.3 kb, and is available from Genbank as bioproject PRJNA495862.

### 2.4 Species confirmation and amplification of gene fragments

Prior to analysis, the identity of species was confirmed by amplification of an 1.8 kb fragment of the SSU rDNA from genomic DNA using primers 18SF-A and 18SR-B (Medlin et al., 1988) and 2x MyTaq™ Red Mix (Bioline) Taq polymerase. Denaturing of gDNA for 3 min at 95 °C was followed by 35 amplification cycles of 45 s at 55 °C, 60 s at 70 °C and 30 s at 95 °C with final extension for 5 min at 70 °C. Amplified fragments were analysed by agarose gel electrophoresis and cloned into pGEM®-T Easy (Promega) or pCR™4-TOPO® TA (Thermo Fisher Scientific) cloning vectors and sequenced with M13F and M13R primers. Where SSU rDNA sequence was not distinctive enough for species diagnosis, the internal transcribed spacer (ITS) was also amplified using oligonucleotide primers ITS1 and ITS2 (Romeralo et al., 2007), and cloned and sequenced as described above.

Fragments of the test genes ranging from 0.4 kb to 1.6 kb were amplified using degenerate primers, designed complementary to well-conserved regions (See Appendix A, Table A2). A two-step amplification program was used with 10 cycles of 45 s at 50–52 °C, 0.5–2 min at 70 °C and 30 s at 95 °C, followed by 25 cycles of 45 s at 52–54 °C, 0.5–2 min at 70 °C and 30 s at 95 °C, with initial denaturation and final extension as above. For some fragments that failed to amplify, the extension temperature was lowered from 70 °C to 64 °C. Cloning and sequencing was the same as for the 18S SSU and ITS fragments. To account for PCR and sequencing errors at least two independent clones were sequenced and only entered into the analysis if they showed complete agreement at the amino acid level.

### 2.5 Gene retrieval from newly sequenced and published genomes.

Draft genome assemblies for *D. citrinum*, *D. intermedium* and *D. firmibasis* were obtained from NCBI with accession numbers PRJNA45877, PRJNA45879 and PRJNA45875, respectively. Illumina transcriptome reads from *D. giganteum* were retrieved from the DNA databank of Japan (DDBJ) with accession number SRX020186. Raw reads were trimmed and assembled into contigs with CLC Genomics Workbench 9.5.3 (www.qiagenbioinfromatics.com).

Homologues of the full set of the previously analyzed 47 genes were retrieved by tBLAST search from these genomes/transcriptomes and from the *P. multicystogenum* and *D. caveatum* genomes. Gene models were manually predicted, assisted by alignments of orthologous protein sequences.

### 2.6 Sequence alignment and phylogenetic inference

DNA sequences of *agl*, *amdA, purD, purL, rpaA*, and *smdA* genes were obtained with degenerate PCR and BLAST query of the *D. citrinum*, *D. giganteum*, *D. firmibasis*, *D. intermedium*, *D. caveatum* and *P. multicystogenum* genomes and transcriptomes. Amino-acid sequences were predicted using CLC workbench and introns were assigned manually, assisted by alignment to orthologous protein sequences. A total of 299 amino-acid sequences were individually aligned with their orthologues from the 12 previously analysed *Dictyostelium* genomes and genomes from the non-Dictyostelid Amoebozoa *Physarum polycephalum*, *Protostelium aurantium* var. *fungivorum* and *Acanthamoeba castellani* using ClustalOmega (Sievers and Higgins, 2014) with five combined iterations*.* Sections of sequence with poor consensus alignment and indels in individual or multiple sequences were deleted across the entire alignment using Bioedit (Hall, 1999). Concatenation of the individual alignments of fragments from all six genes yielded an alignment with 2711 positions.

The 47 genes used in the earlier phylogeny of 12 species (Singh et al., 2016) were aligned with their orthologues in the genomes listed above. After concatenation, the alignment measured ∼38951 positions. Phylogenies were inferred using (1) Phylobayes MPI with a CAT + GTR model (Lartillot et al., 2013) with two MCMC chains generated and run for 10,000 cycles, (2) MrBayes 3.2 with partitioning of the alignment into its individual proteins or PCR fragments and each partition run under its most likely amino acid substitution model or (3) RAXML as further outlined in the figure legends. Trees were drawn using Figtree (http://tree.bio.ed.ac.uk/software/figtree/) and rooted on the outgroup of solitary Amoebozoa.

### 2.7 Ancestral state reconstruction

An R package “phytools” was used for ancestral trait reconstruction (Revell and Graham Reynolds, 2012). For continuous traits, the “fastAnc” function implemented in phytools was used to estimate the ML ancestral states for internal nodes. The function returns point estimates as well as variance and 95% confidence intervals for each node. For discrete traits, the “rerootingMethod” function based on (Yang et al., 1995) was used to reconstruct ancestral traits. This method returns posterior probabilities of ancestral states for internal nodes, as well as marginal likelihood at the root. The likelihoods were then compared for the same trait estimated using two different phylogenetic trees. All investigated traits are listed in Supplementary Data 6 Traits.

## 3 Results and discussion

### 3.1 A genome-based core phylogeny of 20 species

**Fig. 1:**
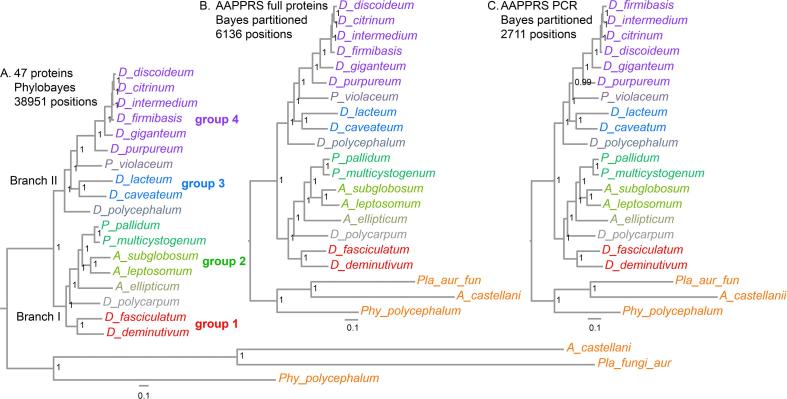
Phylogenies inferred from 47 and 6 proteins from sequenced genomes. *A.47 proteins.* Orthologous sequences of 47 proteins involved in a broad range of cellular functions (Singh et al., 2016) were aligned using Clustal Omega (Sievers and Higgins, 2014). Alignments were edited to remove non-consensual segments and gaps across multiple sequences. After concatenation, the 38,942 AA alignment was subjected to phylogenetic inference using Phylobayes MPI (Lartillot et al., 2013) with a CAT-GTR model. Two MCMC chains were run for 10,000 cycles, with trees sampled at every 10 cycles. The Bayesian consensus tree was generated with a burnin of 1000 cycles. *B.6 full proteins*. Alignments of agl, amdA, purD, purL, rpaA and smdA (AAPPRS) protein sequences, which individually either fully reproduced the earlier 12 species core phylogeny (Singh et al., 2016) or with a single non-consensual node, were concatenated, edited and analysed using MrBayes 3.2. The 6136 AA alignment was partitioned into its individual proteins with each partition run under its most likely amino-acid substitution model over 1 million generations. *C.PCR products.* Sequences of the same six proteins equivalent to the PCR products amplified from 34 test species (Fig. 2) were aligned, concatenated to a total of 2711 AA and analysed as for panel B. Posterior probabilities of the nodes are shown in all trees.

Since completion of the first 47 protein phylogeny of 12 clade-representative species, six more dictyostelid genomes have become available as well as the genome of the protostelid *Protostelium aurantium* var. *fungivorum*, a relatively close outgroup species to Dictyostelia (Hillmann et al., 2018). For each novel genome, we were able to retrieve orthologs of the majority of the earlier 47 proteins by BLASTp or tBLASTn and after gene model prediction, we aligned the deduced protein sequences with those of the 12 Dictyostelia analysed earlier and the amoebozoan outgroup species *Acanthamoeba castellani* and *Physarum polycephalum* (see Supplementary Data 5 Sequences and alignments, sheets 2 and 3) The 47 alignments were concatenated (Supplementary Data 1 47 aligned prot. 21) and used for phylogenetic inference by Phylobayes MPI (Lartillot et al., 2013) (Fig. 1). All methods yielded a single highly supported tree, which, like the earlier multigene phylogenies (Romeralo et al., 2013, Sheikh et al., 2015, Singh et al., 2016), divided all Dictyostelia into two major branches, containing groups 1 and 2 and groups 3 and 4 respectively. The inclusion of a second clade 2B species consolidates the position of *A. ellipticum* as a sister species to the clade uniting clade 2B and the remaining acytostelids in clade 2A, as well as the position of *D. polycarpum* as earliest diverging species of group 2. *D. polycephalum* is consolidated as the earliest diverging species of branch I, thus forming the sister group to the clade combining groups 3 and 4. *D. caveatum* has taken up position as the earliest diverging species of group 3, while *P. violaceum* takes this role for group 4. *D. citrinum*, *D. firmibasis* and *D. intermedium* group closely together with *D. discoideum*, while *D. giganteum* and *D. purpureum* are more distantly related.

### 3.2 Selection of genes for phylogenetic inference

The genes for the first 47 gene core phylogeny were selected to represent a broad range of cellular functions to average out any mutations perpetuated by taxon-specific selection events (Singh et al., 2016). We also selected relatively long genes, which after validation, could be used for classification of species in a PCR approach in addition to the commonly used SSU rDNA. For validation, we assessed the extent to which trees inferred from single genes reproduced the 47 gene core phylogeny of twelve species. Two genes, *rpaA* and *smdA,* fully reproduced the core phylogeny, while trees from another 12 genes contained a single non-consensual node. When two of such genes with different errors were concatenated, they again yielded the consensus phylogeny (Singh et al., 2016). To be suitable for amplification by PCR, the genes must contain well-placed conserved regions that enable design of primers with moderate degeneracy and amplification of sufficiently long stretches of DNA with a large proportion of variable sites. We initially identified seven genes: *aco1, agl, amdA, purD, purL, rpaA* and *smdA.* Because full-length amplification of very large DNA fragments with degenerate primers is often problematic, the amplification targets for *aco1, agl, amdA, purL and smdA* were broken up into four (*aco1)* or two smaller fragments each. Despite our efforts, it was not possible to amplify *aco1* fragments from most of the species. Generally, for the less conserved genes more degenerate primers had to be designed for the different taxon groups than for genes with higher conservation between species. The concatenated sequence of the six selected proteins (Supplementary data 2 AAPPRS_full_align.21) also robustly reproduced our current more extensive 47 protein phylogeny (Compare Fig. 1A and B). The total amplifiable sequence of the 6 genes was only 44% of their total length, but this alignment (Supplementary data 3 AAPPRS_PCReq.21) still reproduced the 47 protein phylogeny (Fig. 1C), except that the relationships between *D. discoideum* and its very close relatives *D. citrinum*, *D. intermedium* and *D. firmibasis* were slightly altered. This validates the use of these genes for phylogenetic inference.

### 3.3 A 6-protein phylogeny of 52 *Dictyostelia*

**Fig. 2:**
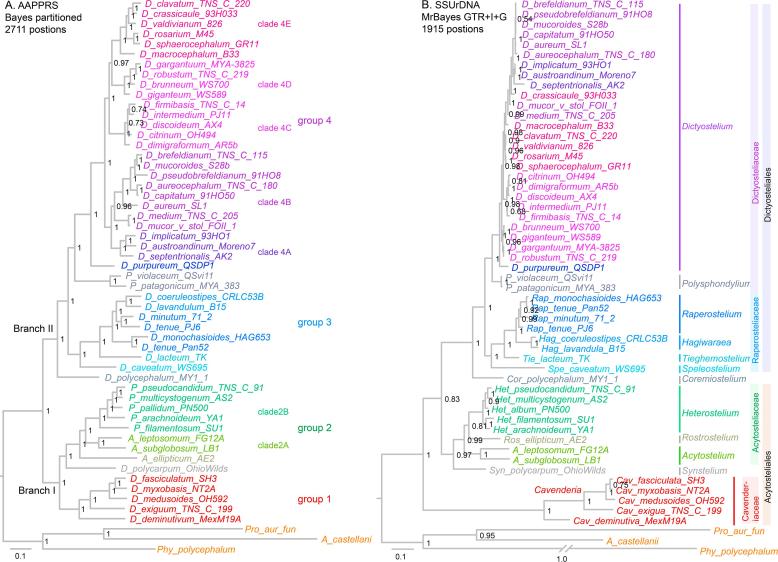
A 6 protein phylogeny of 52 *Dictyostelium* species. *A. 6 proteins.* Fragments of the *agl*, *amdA*, *purD*, *purL*, *rpaA*, *smdA* genes were amplified by PCR from 34 *Dictyostelium* species and retrieved from genome sequences of 18 other Dictyostelia and 3 outgroup amoebozoan species (see Fig. 1) The deduced amino acid sequences were aligned with ClustalOmega and the alignments were concatenated. A phylogeny was inferred using MrBayes 3.2 with the alignment partitioned into its amplified segments. Each segment was analysed over 1 million generations with its most likely amino acid substitution model and a gamma distribution of substitution rates. Taxon names follow those of the original species diagnoses. *B. SSU rDNA.* SSUrDNA sequences for all 55 species were retrieved from Genbank, aligned with mCoffee and subjected to Bayesian inference using a GTR model with a gamma distribution and a proportion of invariable sites over 1 million generations. Taxon names follow the recently proposed re-classification of Dictyostelids (Sheikh et al., 2018). Note that the genus *Raperostelium* in the SSU rDNA tree is paraphyletic in the AAPPRS tree.

The goal of the current study was to resolve the relationships between species within group 4 and the relationship between group 4 and the other Dictyostelia. We therefore incorporated 27 species from group 4, six from group 3 and three from groups 1 and 2 each into the new phylogeny, in addition to the 18 species with sequenced genomes, that are described above. In total, we amplified and sequenced 299 gene fragments from 34 species of Dictyostelia (Appendix A, Table A1). However, for some species, some fragments defied amplification even with alternatively designed primer sets. The deduced amino acid sequences from the protein fragments were aligned and concatenated to a total length of 2711 positions (see Supplementary Data 5 Sequences and alignments, sheets 5 and 6 and Supplementary data 4 AAPPRS_PCRprod.55). A phylogeny was inferred using MrBayes 3.2 with the alignment partitioned into its PCR fragments and each fragment analysed with its most likely amino acid substitution model (Fig. 2A).

As was the case with the 47 protein phylogeny (Fig. 1), the 6 protein phylogeny, further called the AAPPRS phylogeny after the acronym of its 6 protein names, subdivided Dictyostelia into two major branches: branch I, recently reclassified as Acytosteliales (Sheikh et al., 2018), containing group 1 (Cavenderiaceae) and group 2 (Acytosteliaceae) and branch II (Dictyosteliales) comprising group 3 (Raperosteliaceae) and group 4 (Dictyosteliaceae). *D. polycarpum* is also here the earliest diverging species of group 2 and *A. ellipticum* second and sister species to both clade 2A (*Acytostelium*) and clade 2B (*Heterostelium*).

*D. polycephalum* is a sister species to both groups 3 and 4 in branch II, while *D. caveatum* is either a sister species to or member of group 3, with *D. lacteum* as closest relative. The remaining six species separate into three clades of two, with the two crampon-based species *D. coeruleostipes* and *D. lavandulum* grouping together. As with the earlier phylogenies, the “violaceum” complex, now the genus *Polysphondylium* and containing *P. violaceum* and *P. patagonicum*, remains the closest outgroup to group 4. Interestingly, *D. purpureum*, which shares its purple colour with *P. violaceum and P. patagonicum,* is its closest relative, despite being otherwise phenotypically quite distinct (Romeralo et al., 2013). The remaining species in group 4 separate into two divisions, which tentatively separate into two clades 4A and 4B for the bottom division and three clades 4C-E in the top division. This tentative arrangement may however become more elaborate when more species are included in future. Inference of the same alignment by RAXML or without the outgroup species yielded the same overall phylogeny with only a few changes in the relative positions of very closely related group 4 species (Appendix A, Fig. A1).

When comparing the AAPPRS phylogeny with an SSU rDNA phylogeny of the same species (Fig. 2B), the most striking difference is that branch lengths across the tree are much more uniform in the AAPPRS phylogeny. Particularly, the very deep branch that separates the group 1 (Cavenderia) from the other groups in the SSU rDNA tree is not present in the AAPPRS phylogeny. Conversely, the very short branches that preclude proper resolution between many group 4 species in the SSU rDNA tree are considerably longer in the AAPPRS tree. Two of the group 4 clades in the AAPPRS tree can also be recognized in the SSU rDNA tree, but their order relative to the outgroup (*P. violaceum* and *P. patagonicum)* is different, while species of the other clades are intermixed.

The AAPPRS tree also differs from the SSU rDNA tree in the order of some clades in group 3, which were assigned the rank of genus in the recent reclassification (Sheikh et al., 2018). While we consider a separate genus status justified for the *Hagiwaraea,* which contains all crampon-based species (Schaap et al., 2006, Sheikh et al., 2018) (here *D. coeruleostipes* and *D. lavandulum)*, it is not obvious why the other clades deserve this distinction. In the AAPPRS tree the new genus *Raperostelium* also appears to be polyphyletic, although this conclusion is drawn from only few tested species. The other differences between the AAPPRS and SSU rDNA trees are the position of the root – between branch I Acytosteliales and branch II Dictyosteliales in the AAPPRS tree and between group 1 (Cavenderiaceae) and group 2 (Acytosteliaceae) in the SSU rDNA tree. The latter position is however unstable and was not found with the same alignment analysed by RAXML (Fig. A1B). Earlier SSU rDNA trees are even less consensual with the AAPPRS tree and show *D. polycarpum* as sister species to *D. polycephalum* (Schaap et al., 2006, Sheikh et al., 2018) and *D. purpureum* as sister to *D. macrocephalum* (Schaap et al., 2006). We tried to emulate the most recent SSU rDNA tree (Sheikh et al., 2018) by using the same alignment and inference methods and parameters, but while this moved *D. purpureum* closer to *D. macrocephalum*, it did not change the position of *D. polycarpum.* Our SSU rDNA alignment was less stringently edited (1915 instead of 1560 positions), which may have retained more phylogenetic signal to discriminate between *D. polycephalum* and *D. polycarpum* (Fig. A1C)*.*

### 3.4 Trait mapping to and ancestral state reconstruction from alternative tree topologies

We previously measured and mapped 25 phenotypic characters to the then available SSU rDNA phylogeny of 99 *Dictyostelium* taxa (Romeralo et al., 2013, Schilde et al., 2014). Phylogenetic comparative methods highlighted some trends in phenotypic evolution of Dictyostelia, which will not be the same if the underlying phylogeny has a different topology. A visual representation of the earlier traits mapped to either the AAPPRS or SSU rDNA phylogeny (Appendix A, Fig. A2) does not show any striking differences. As also recorded with the earlier genome based phylogeny of 12 species, G/C content is lower in branch II than branch I, with the lowest G/C content over 6 amplified coding regions found in the group 4 species *D. medium* (27%) and the highest in the group 2 species *A. ellipticum* (61%). Some features such as large aggregates, sori and stalks, freely migrating slugs and cellular supports for the stalk, which in the SSU rDNA phylogeny evolved in the earliest diverging group 4 taxa and were then lost again in the late diverging taxa, are in the AAPPRS phylogeny associated with one, but not the other, major division of group 4. This seems a more likely scenario, since it does not require a secondary loss.

**Fig. 3:**
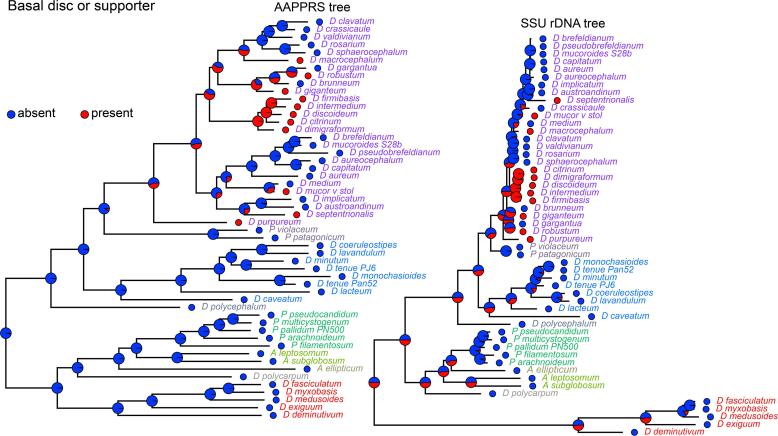
Ancestral state reconstruction. The trait “stalk supports” was reduced to a binary expression (see Supplementary Data 6 Traits) and trait evolution was examined over both the AAPPRS and SSUrDNA tree using the “rerootingMethod” implemented in phytools (Revell, 2012) Small circles next to species names are colour coded to represent the presence (red) or absence (blue) of the trait, while the fractional colour coding in the larger circles represents the posterior probabilities of the trait at the internal nodes. (For interpretation of the references to colour in this figure legend, the reader is referred to the web version of this article.)

As illustrated in Fig. 3, the large differences in SSU rDNA evolution rates between groups, as evident from the very short and long branch-lengths in groups 4 and 1, respectively, lead to some striking differences during inference of ancestral states by bayesian and maximum likelihood based methods. Both methods incorporate both the internal node topology and the branch lengths (reflecting the time span since the taxa started to diverge) in these computations. For a trait like stalk support that only evolved in group 4, the short branch lengths of group 4 result in this trait still being assigned with 50% probability in the last common ancestors (LCAs) of groups 1, 2 and 3 in the SSU tree (Fig. 3B), while in the AAPPRS tree, this probability is zero (Fig. 3A). Ancestral state reconstruction (ASR) across different phylogenies can also indicate which tree topology provides the most likely scenario of trait evolution. We performed such an analysis for all continuous (quantitative) and discrete (qualitative) traits shown in Fig. A2. All visual representations of ASRs are shown in Appendix A, Figs. A3 and A4 for continuous and discrete traits, respectively. For discrete traits, the marginal likelihood at the root represents a measure of the goodness of fit of the tree to the traits (Table 1). The methods available for ancestral state reconstruction of continuous traits do not return such a value. Here we used the averaged variance of the trait estimates at all interior nodes (Table 1) (Supplementary Data 7 AncestralStates, sheets 3–5). For 7 out of 13 discrete traits the AAPPRS tree results in a more likely evolutionary history than the SSU rDNA tree, for the remaining 6 traits the SSU rDNA tree is more likely. The log likelihood differences were at 3.44 on average larger for the 7 ASR’s favouring the AAPPRS than the 6 ASRs favouring the SSU rDNA tree (1.06). For the 12 continuous traits averaged variances of ancestral states are on average four-fold lower for the AAPPRS tree than the SSU rDNA tree. It therefore appears that overall the AAPPRS tree provides the basis for more robust statistical inference of ancestral traits than the SSU rDNA tree.

**Table 1:** Tree validity tests from ancestral state reconstruction.

Discrete trait	AAPPRS Log likelihood	SSU_rDNA Log likelihood	Continuous trait	Mean variance ratio AAPPRS/SSU rDNA
Stalk support	**−28.22**	−31.18	Spore diameter	0.22
Polar granules	−16.97	**−15.56**	Spore eccentricity	0.21
Aggregation	−15.10	**−13.76**	Amoeba diameter	0.23
Habit	−19.21	**−18.88**	Amoeba eccentricity	0.13
Sorocarp branching	**−27.15**	−29.74	Aggregate diameter	0.21
Lateral branches	**−25.01**	−32.30	Sorogen length	0.54
Regular whorls	**−9.22**	−9.69	Sorus diameter	0.26
Pointed stalk tip	**−19.81**	−24.16	Stalk area	0.21
Broadened_tip	**−23.75**	−27.59	Stalk eccentricity	0.39
Stalked_migration	**−**33.67	**−30.27**	Anterior prestalk	0.30
Free_migration	**−17.23**	−19.81	Posterior prespore	0.19
Phototropism	**−**23.78	**−23.03**	Rearguard region	0.15
Microcyst	−18.99	**−17.65**		

Discrete traits were converted to binary expressions (Supplementary Data 6 Traits) and subjected to ancestral state reconstruction over the AAPPRS and SSU rDNA phylogenies (Fig. A4) using the “rerootingMethod” function implemented in phytools (Revell, 2012). The “goodness of fit” of trait evolution to the tree, as represented by the marginal log likelihood at the root is presented for each trait. The lower values (in bold) represent the best fit for each trait. For continuous traits ancestral state reconstruction was performed with “fastAnc” in phytools, which returns node estimates with variances and 95% confidence intervals (see Supplementary Data 7_AncestralStates). The variances for each trait were averaged here and the ratio of averaged variances obtained with AAPPRS tree over those obtained with the SSU rDNA tree was calculated. All variances were lower for ancestral trait estimates in the AAPPRS tree.

### 3.5 Single gene trees

The availability of more sequenced genomes allowed us to re-assess the suitability of individual proteins for phylogenetic inference by investigating the extent to which trees inferred from individual proteins reproduced the consensus 47 protein phylogeny. All 47 individual protein trees are shown in Appendix A, Fig. A5, annotated with the number of aligned positions and non-consensual nodes. Only seven of the single protein trees reproduced the 47 protein core phylogeny exactly, and many trees showed from one to up to four deviations from the consensus and/or were poorly resolved. Two of our test proteins, smdA and rpaA, performed worse than before (Table 2), and some proteins, such as DDB_G0270990, DDB_G0271904, DDB_G0289993, rpa2 and rpc3 performed better, possibly due to improved alignment with the larger number of taxa. In practical terms, *smdA* was also less suitable because only small regions of DNA could be amplified, due to the close spacing of conserved regions suitable for primer design. Overall, the comparison of single gene trees shows that our current set of proteins for phylogenetic inference can still be refined and that a smaller set of 2 or 3 proteins that complement each other’s errors may suffice for correct classification of *Dictyostelium* species.

**Table 2:** Performance of single protein phylogenies.

Number of non-consensual nodes									
0		1		2		3		4	
Protein	AA	Protein	AA	Protein	AA	Protein	AA	Protein	AA
aco1(1)	882	accA(3)	2183	alxA (2)	699	5NT(3)	516	aclY(2)	610
agl (1)	1474	amdA (1)	678	cinC (2)	837	acsa (2)	653	clcD (2)	825
270990(1)	601	glnA3(1)	732	287723(2)	912	argC (2)	740	grpA	211
271904(1)	604	midA(1)	422	290197(2)	496	cas1 (2)	699	scdA(3)	666
289993(1)	613	pdhC(1)	497	glpD(2)	878	dcsA (3)	975		
rpa2(2)	1130	purD(1)	787	glud2(3)	1004	276321(2)	506		
rpc3(2)	567	purL(1)	1325	hdaB(2)	391	dnmA (3)	314		
		pyr13(1)	2144	ogdH(3)	975	fcsA(2)	646		
		rpa1(0)	1460	pfkA(2)	765	gcsa(3)	602		
		rpb1(2)	1541	pgmB(4)	577	glpV(2)	852		
		rpc1(2)	1372	sglA(3)	526	sdhA(3)	611		
				smdA(0)	402				
				tkt2(4)	661				
				xdH(3)	1318				

Summary of the number of non-consensual nodes detected in 21-species phylogenies inferred from the individual proteins of the 47 protein phylogeny (see Appendix A, Fig. A5). Data in parentheses are the number of non-consensual nodes detected in the earlier 14-species phylogenies of the same proteins (Singh et al., 2016). The proteins used in the AAPPRS phylogeny are underlined. The DDB_G0 prefixes of genes with 12 character gene names are not shown.

## 4 Conclusions

We firstly used a concatenated set of 47 functionally divergent orthologous proteins from 21 sequenced genomes to expand the core phylogeny of Dictyostelia and three outgroup Amoebozoa. The new phylogeny consolidates the position of the root to Dictyostelia between two branches each containing two major groups. It robustly positions *D. polycarpum* and *P. violaceum* as sister groups to groups 2 and 4 respectively, as well as *D. polycephalum* as sister to groups 3 and 4, and *A. ellipticum* as sister to clades 2A and 2B.

We amplified sequences encoding a set of six proteins (Agl, AmdA, PurD, PurL, RpaA, SmdA), which, when concatenated, robustly reproduced the core phylogeny over 34 *Dictyostelium* species. Our main goal was to investigate species relationships within group 4, which were poorly resolved in the earlier SSU rDNA phylogeny and to elaborate the core phylogeny with 3–5 more species in each taxon group. The new AAPPRS phylogeny separates group 4 into two subgroups, which further partition into five clades. This topology differs radically from that of the SSU rDNA phylogeny, where separate clades, when recognizable are nested inside one another.

In the course of Dictyostelid evolution the most dramatic phenotypic innovations occurred in group 4 with the acquisition of cAMP as attractant for aggregation, an overall increase in the size of aggregates and fruiting bodies, the appearance of three novel somatic cell types and the ability to pre-specify cells for either spores, stalk or other support structures, rather than de-differentiating prespore cells to form the stalk. The new phylogeny presents a robust framework for investigating the order in which these events and their underlying regulatory mechanisms evolved.

While eventually a gold standard phylogeny may be obtained from whole genome or transcriptome sequencing of all Dictyostelia, the current approach of PCR amplification of a few well-validated genes provides a low cost alternative for accurate classification of existing and newly isolated species.

## Funding

This work was supported by the European Research Foundation [742288], the Wellcome Trust [100293/Z/12/Z], the European Molecular Biology Organisation [ALTF 295-2015] and the Japanese Organisation for the Promotion of Science [H28-1002].

## Data availability

All sequences amplified in the course of this work are listed in Supplementary Data 5 Sequences and alignments. The *P. multicystogenum* and *D. caveatum* genome sequences have been submitted to Genbank under Bioproject IDs PRJNA495730 and PRJNA495862, respectively.

## Author contributions

CS and HL amplified DNA sequences, CS inferred gene models and aligned protein sequences, CS, PS and KK performed phylogenetic inference, KK inferred ancestral traits and assembled the *P. multicystogenum* genome, ISH and KI sequenced the *D. caveatum* genome and CS and PS wrote the manuscript.

## Conflict of interest

None.

## Appendix A and Supplementary data files

Appendix A. Supplementary figures and tables

Supplementary Data 1 47 aligned prot. 21

Supplementary Data 2 AAPPRS_full_align.21

Supplementary Data 3 AAPPRS_PCReq. 21

Supplementary Data 4 AAPPRS_PCR prod. 55

Supplementary Data 5 Sequences and alignments

Supplementary Data 6 Traits

Supplementary Data 7 Ancestral States
