# Supplementary material for: A well supported multi gene phylogeny of 52 dictyostelia
Source: Mol Phylogenet Evol. 2019 May;134:66–73. doi: 10.1016/j.ympev.2019.01.017 (PMC6430600; doi:10.1016/j.ympev.2019.01.017)
Supplement: Appendix A. Supplementary figures and tables [file mmc1.pdf]

# APPENDIX A

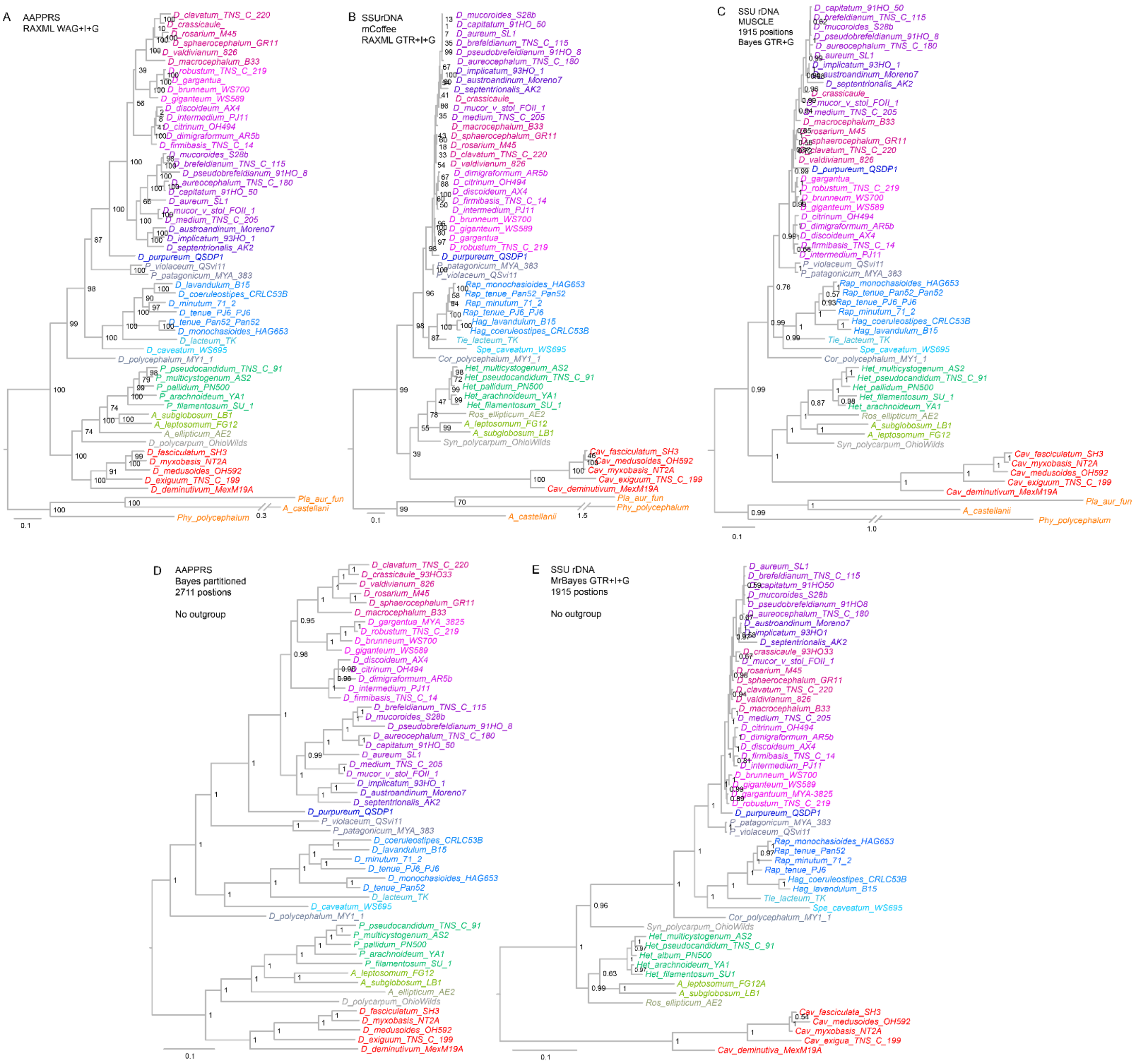


**Figure A1. The AAPPRS and SSU rDNA phylogenies inferred by alternative methods.**
A. The 6 protein (AAPPRS) alignment used for figure 2A was also analysed with RAXML using the preferred WAG+I+G model. Node probabilities were estimated with 100 bootstrap replicates.
B. The mCoffee SSU rDNA alignment of figure 2B was similarly analysed with RAXML with a GTR+I+G model.
C. SSU rDNA sequences were alternatively aligned using MUSCLE with default parameters and analysed by Bayesian inference with a GTR+G model, both as used in (Sheikh et al., 2018).
D/E. The AAPPRS and mCoffee SSU rDNA alignments analysed by Bayesian inference as in figure 2 without the amoebozoan outgroup sequences and rooted at midpoint.

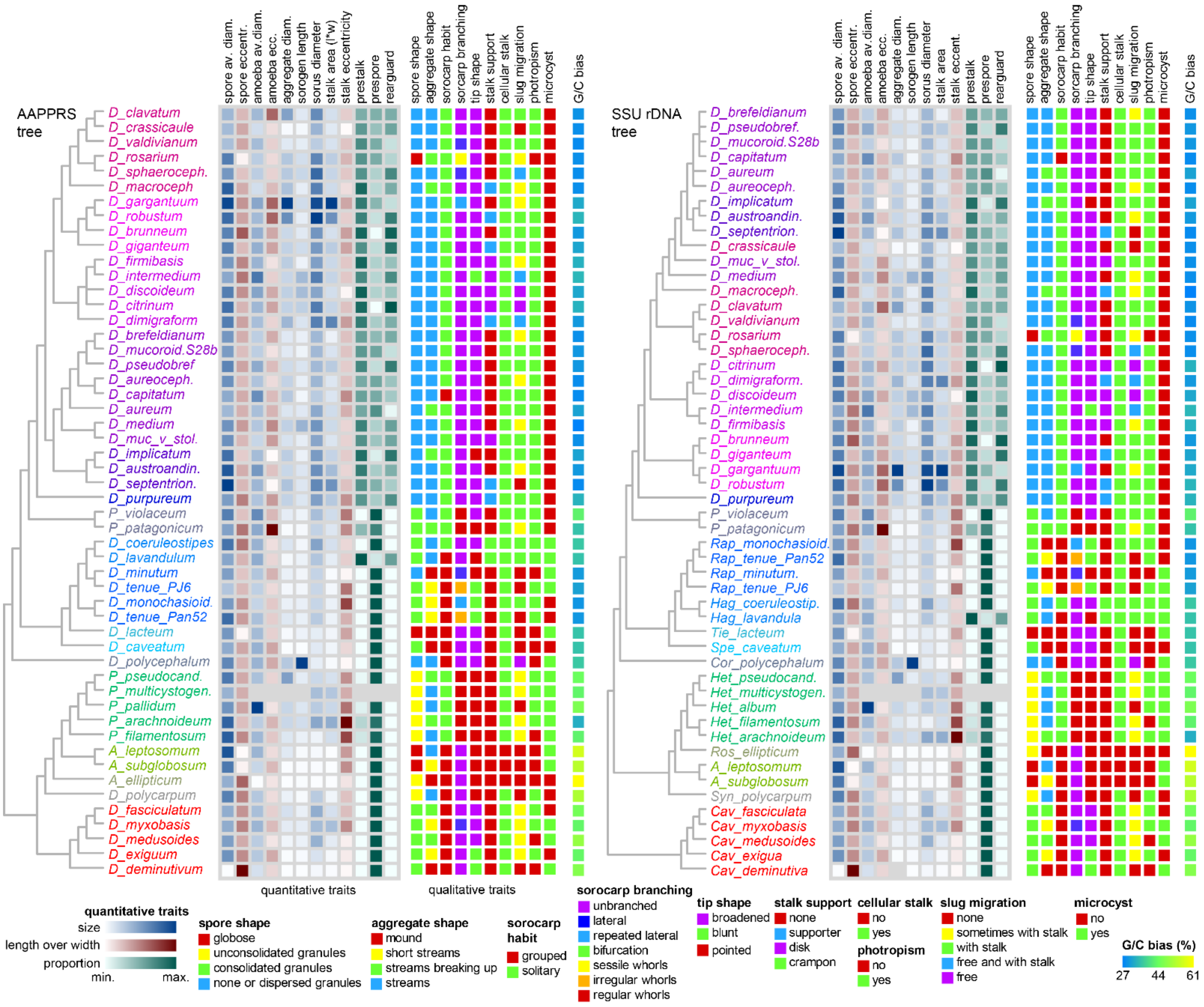


**Figure A2. Mapping of phenotypic traits to the AAPPRS and SSUrDNA phylogenies**
Species traits that were measured previously (Romeralo et al., 2013; Schilde et al., 2014) (Supplemental Data2_Traits.xlxs) were mapped onto the AAPPRS and SSUrDNA phylogenies of figure 2. Traits for *P. multicystogenum* were retrieved from the original diagnosis (Kawakami and Hagiwara, 2008) and G/C bias was determined from the DNA sequences of the PCR products amplified in the course of this work, or from the equivalent regions retrieved from sequenced genomes (Data2_Traits.xlxs, sheet 5).

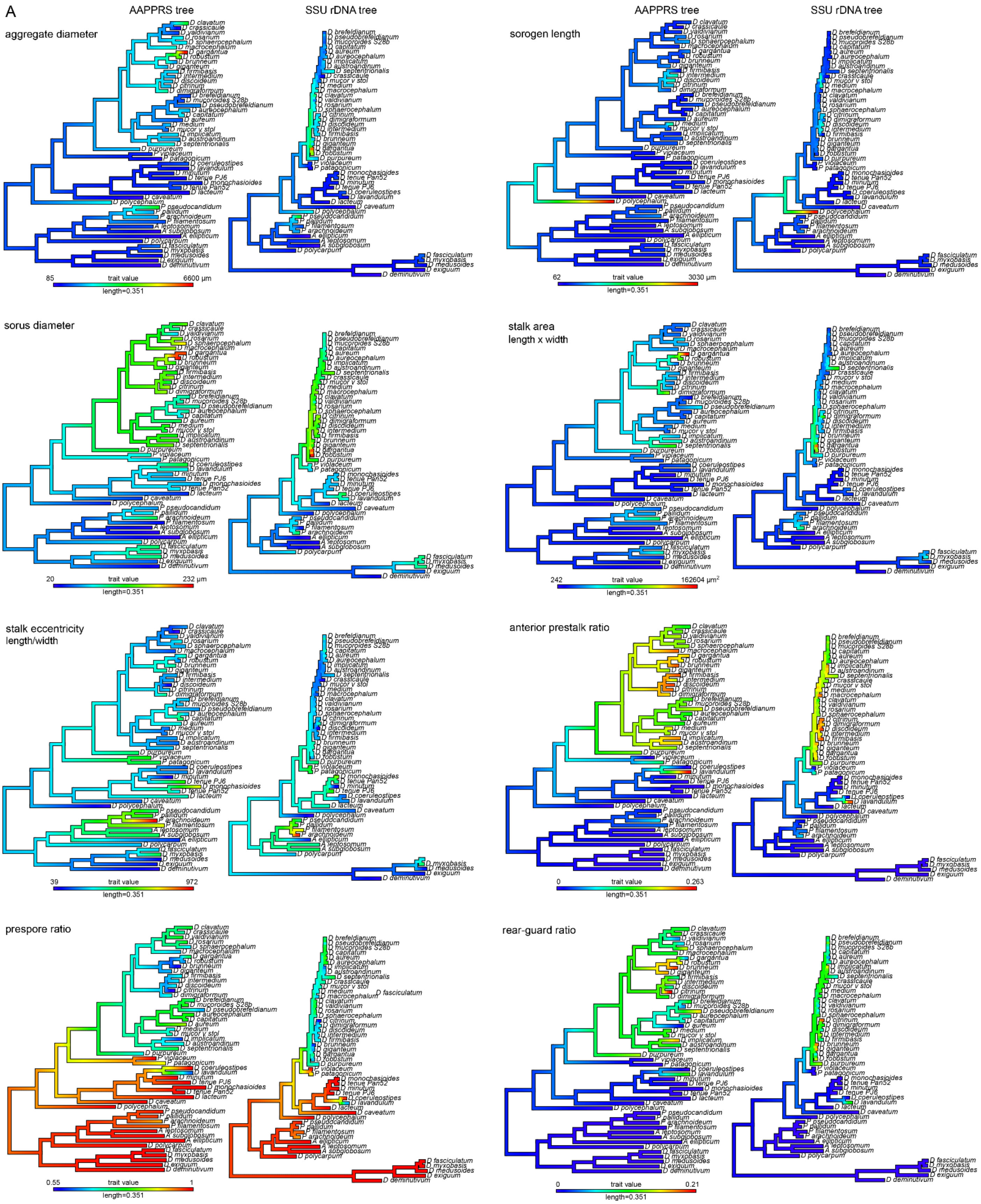
A
AAPPRS tree
SSU rDNA tree
AAPPRS tree
SSU rDNA tree
aggregate diameter
85 trait value 6600 μm
length=0.351
sorogen length
62 trait value 3030 μm
length=0.351
sorus diameter
20 trait value 232 μm
length=0.351
stalk area
length x width
242 trait value 162604 μm²
length=0.351
stalk eccentricity
length/width
39 trait value 972
length=0.351
anterior prestalk ratio
0 trait value 0.263
length=0.351
prespore ratio
0.55 trait value 1
length=0.351
rear-guard ratio
0 trait value 0.21
length=0.351

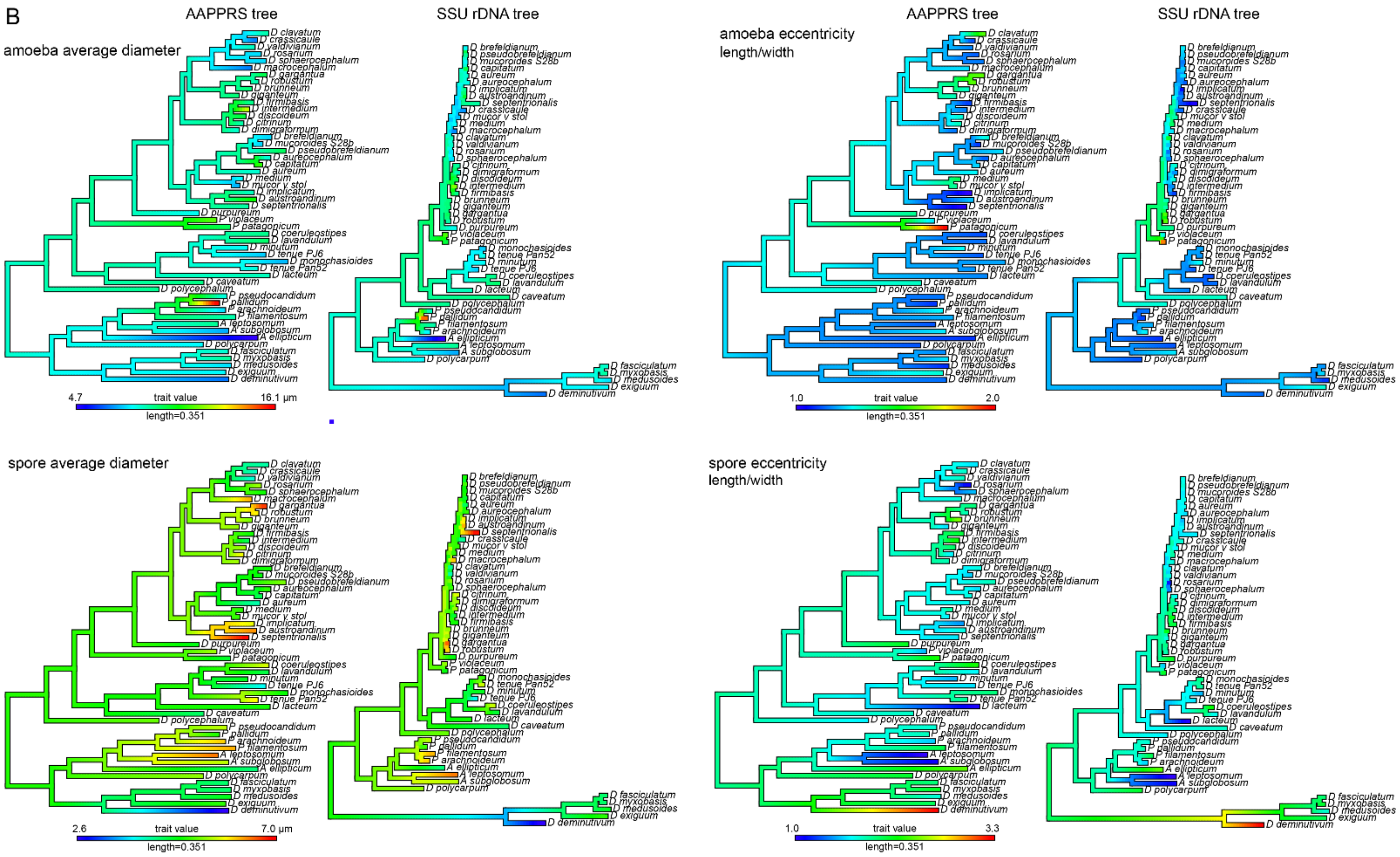


**Figure A3. Ancestral state reconstruction of continuous traits**
Previously measured continuous (quantitative) traits (Romeralo et al., 2013; Schilde et al., 2014) of the multicellular structures (A) or cells (B) were combined with either the AAPPRS or SSU rDNA phylogenies and submitted to ancestral state reconstruction. The “fastAnc” function implemented in the R package “phytools” was used to estimate the maximum likelihood ancestral states for internal nodes, which are here represented by heatmap colour coding. This function returns point estimates as well as variances and 95% confidence intervals for each node (see Data 3_AncestralStates_Quant.xlxs).

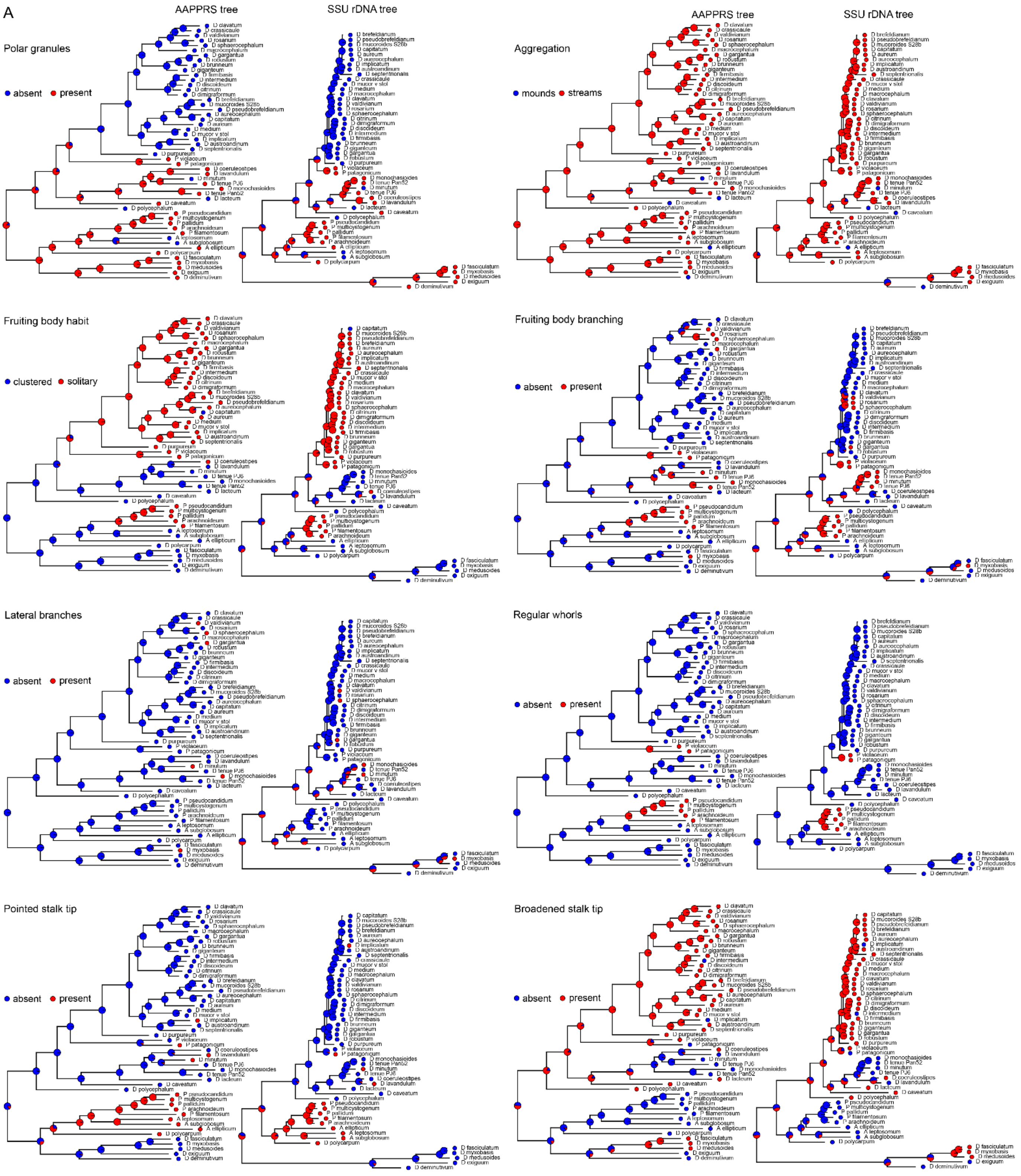
A
AAPPRS tree
SSU rDNA tree
Polar granules
absent present
Aggregation
mounds streams
Fruiting body habit
clustered solitary
Fruiting body branching
absent present
Lateral branches
absent present
Regular whorls
absent present
Pointed stalk tip
absent present
Broadened stalk tip
absent present

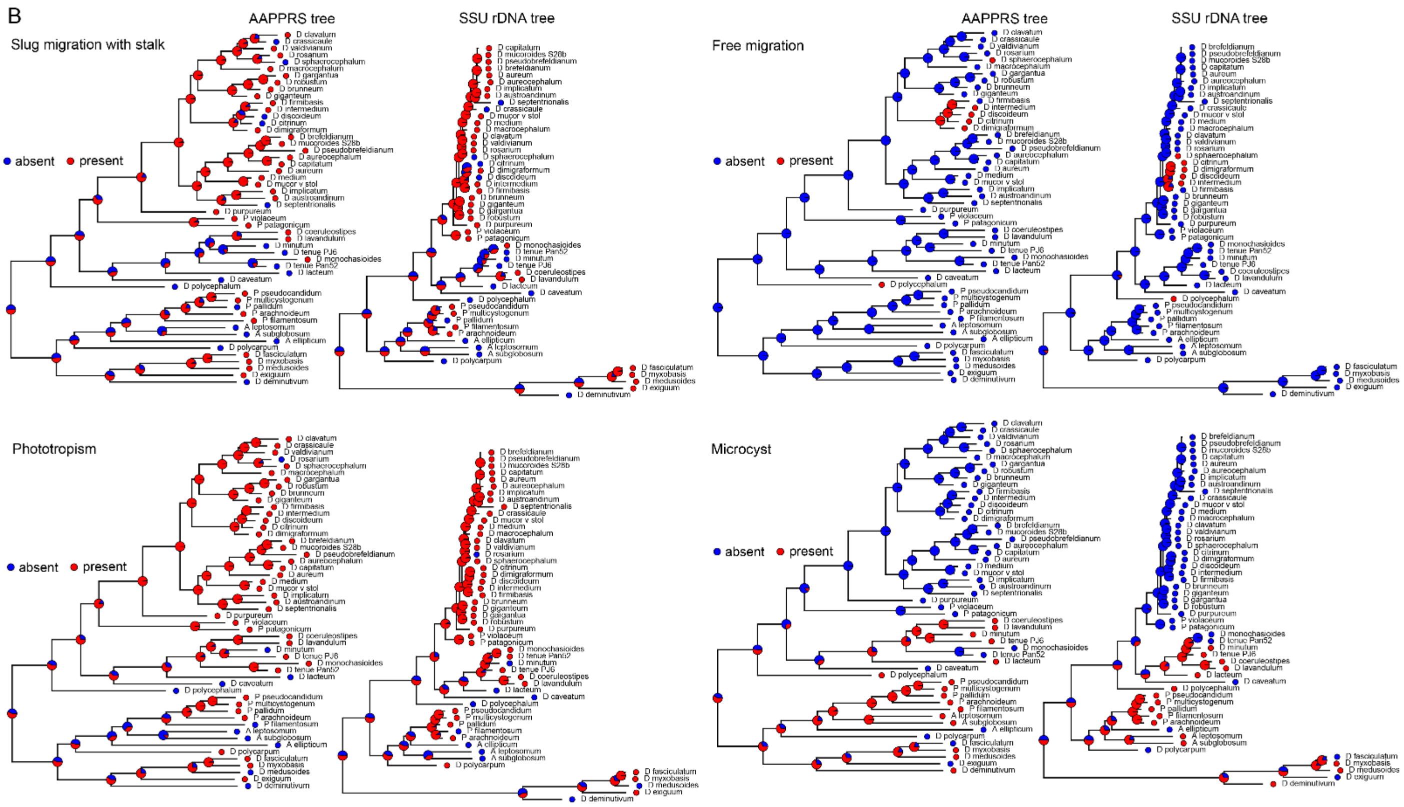


**Figure A4. Ancestral state reconstruction of discrete traits**

A/B. Previously measured discrete (qualitative) traits (Romeralo et al., 2013; Schilde et al., 2014) were converted into binary expressions, mostly expressing the presence or absence of a trait state when there were more than two states of the trait. The data were combined with either the AAPPRS or SSU rDNA phylogeny and submitted to ancestral state reconstruction. The "rerootingMethod" was implemented in phytools (Revell, 2012) was used, which returns posterior probabilities of ancestral states for internal nodes, as well as a marginal likelihood at the root.

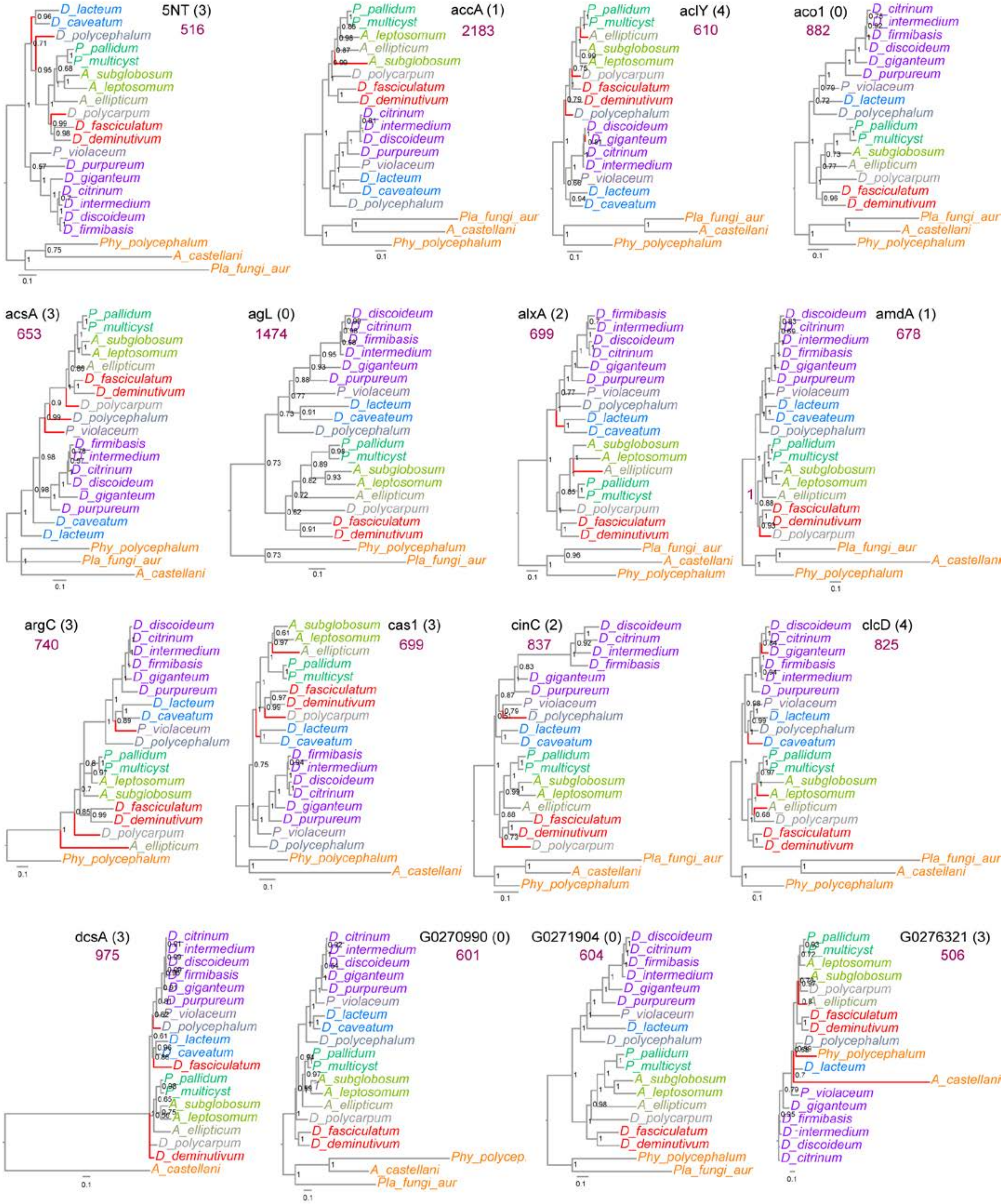

5NT (3)
516
accA (1)
2183
aclY (4)
610
aco1 (0)
882
acsA (3)
653
agL (0)
1474
alxA (2)
699
amdA (1)
678
argC (3)
740
cas1 (3)
699
cinC (2)
837
clcD (4)
825
dcsA (3)
975
G0270990 (0)
601
G0271904 (0)
604
G0276321 (3)
506

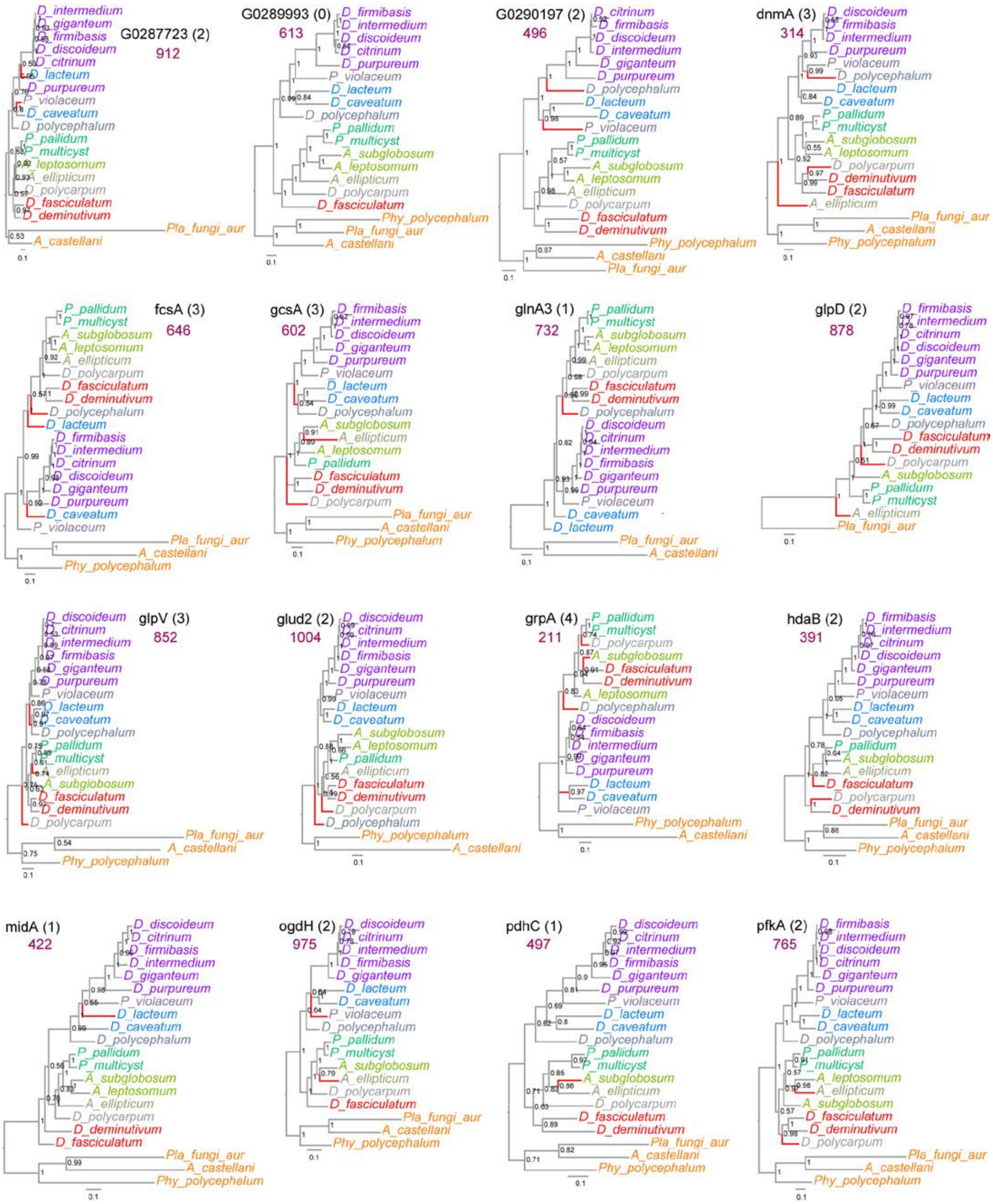
G0287723 (2)
912
G0289993 (0)
613
G0290197 (2)
496
dnmA (3)
314
fcsA (3)
646
gcsA (3)
602
glnA3 (1)
732
glpD (2)
878
glpV (3)
852
glud2 (2)
1004
grpA (4)
211
hdaB (2)
391
midA (1)
422
ogdH (2)
975
pdhC (1)
497
pfkA (2)
765

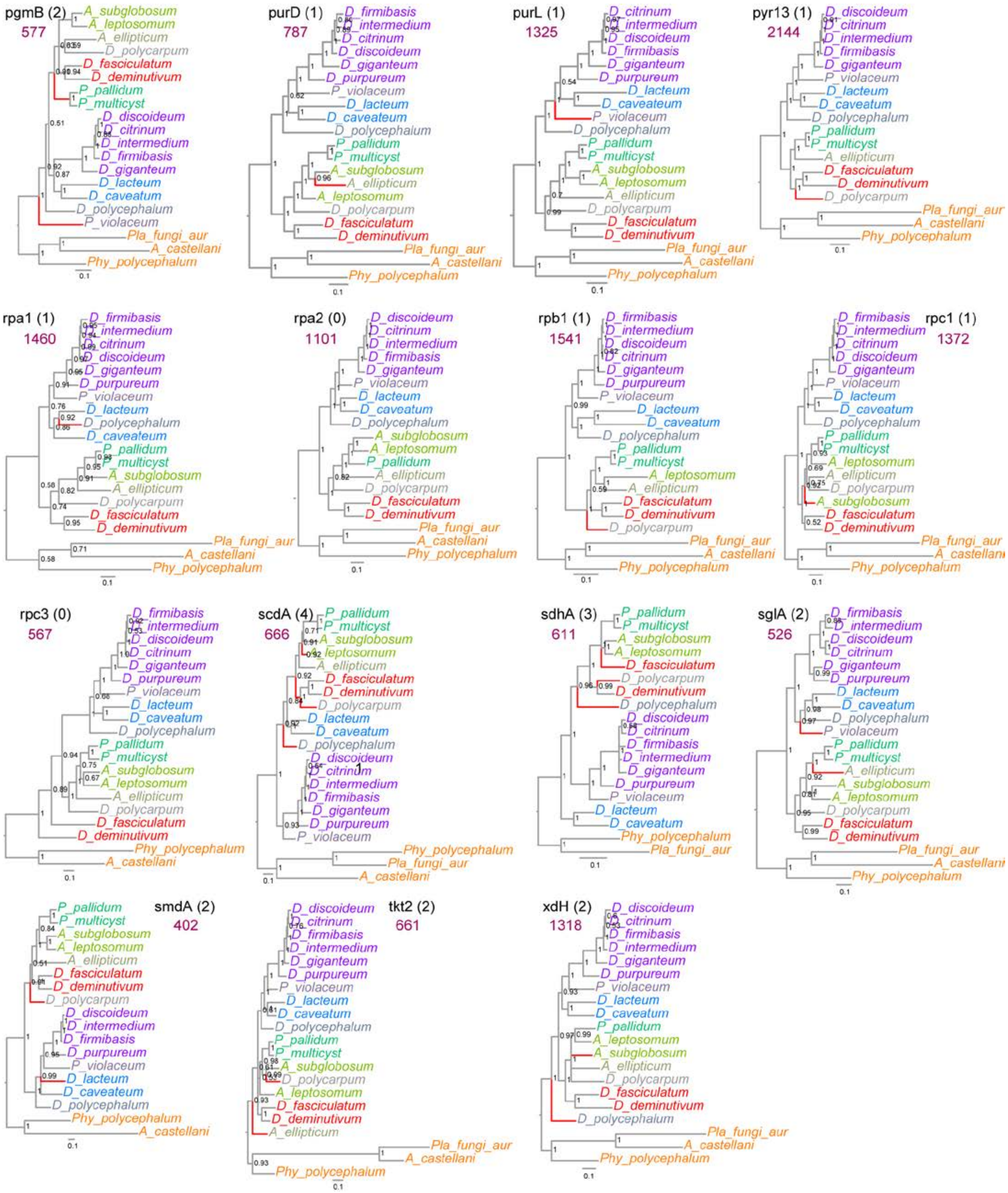


**Figure A5. Single protein trees**

The alignments of the 47 proteins that were concatenated for the phylogeny shown in figure 1A were individually subjected to phylogenetic inference with MrBayes 3.2, using a mixed amino acid substitution model with rate variation between sites estimated by a gamma distribution. Analysis were run for 500.000 generations and trees were rooted on non-Dictyostelid amoebozoa (in amber). Species names were colour coded according to taxon group as in figure 1. The number of aligned positions is indicated in maroon text. Branches that do not conform to the 47 protein phylogeny of figure 1A are shown in red and the number of non-consensual branches for each tree are shown in parenthesis. The order of the very closely related

species *D. discoideum*, *D. citrinum*, *D. firmibasis* and *D. intermedium* (see figure 2) was not included in this count.

**Table A1. *Dictyostelium* species used for PCR amplification**

| Original names | strain | obtained from | new (2018) classification |
|---|---|---|---|
| *D_aureocephalum* | TNS-C-180 | J.C. Cavender | *D_aureocephalum* |
| *D_aureum* | SL1 | J.C. Cavender | *D_aureum* |
| *D_brefeldianum* | TNS_C_115 | H. Hagiwara | *D_brefeldianum* |
| *D_brunneum* | WS700 | J.C. Cavender | *D_brunneum* |
| *D_capitatum* | 91HO50 | H. Hagiwara | *D_capitatum* |
| *D_clavatum* | TNS-C-220 | H. Hagiwara | *D_clavatum* |
| *D_coeruleostipes* | CRLC53B | J.C. Cavender | *Hag_coeruleostipes* |
| *D_crassicaule* | 93H033 | H. Hagiwara | *D_crassicaule* |
| *D_dimigraformum* | AR5b | J.C. Cavender | *D_dimigraformum* |
| *D_exiguum* | TNS-C-199 | H. Hagiwara | *Cav_exigua* |
| *D_gargantuum* | MYA-3825 | J.C. Cavender | *D_gargantuum* |
| *D_implicatum* | 93HO1 | H. Hagiwara | *D_implicatum* |
| *D_lavandulum* | B15 | H. Hagiwara | *Hag_lavandula* |
| *D_macrocephalum* | B33 | H. Hagiwara | *D_macrocephalum* |
| *D_medium* | TNS-C-205 | H. Hagiwara | *D_medium* |
| *D_medusoides* | OH592 | J.C. Cavender | *Cav_medusoides* |
| *D_minutum* | 71-2 | G. Gerisch | *D_minutum* |
| *D_monochasioides* | HAG653 | H. Hagiwara | *Rap_monochasioides* |
| *D_mucoroides* | S28b | J.C. Cavender | *D_mucoroides* |
| *D_mucoroides var_stoloniferum* | FOII-1 | J.C. Cavender | *D_mucoroides var_stoloniferum* |
| *D_myxobasis* | NT2A | J.C. Cavender | *Cav_myxobasis* |
| *D_pseudobrefeldianum* | 91H08 | H. Hagiwara | *D_pseudobrefeldianum* |
| *D_robustum* | TNS-C-219 | H. Hagiwara | *D_robustum* |
| *D_rosarium* | M45 | H. Hagiwara | *D_rosarium* |
| *D_septentrionalis* | AK2 | H. Hagiwara | *D_septentrionalis* |
| *D_sphaerocephalum* | GR11 | J.C. Cavender | *D_sphaerocephalum* |
| *D_tenue* | Pan52 | J.C. Cavender | *Rap_tenue* |
| *D_tenue* | PJ6 | J.C. Cavender | *Rap_tenue* |
| *D_austroandinum* | Moreno 7 | J.C. Cavender | *D_austroandinum* |
| *D_valdivianum* | 826 | J.C. Cavender | *D_valdivianum* |
| *P_arachnoideum* | YA1 | J.C. Cavender | *Het_arachnoideum* |
| *P_filamentosum* | SU1 | J.C. Cavender | *Het_filamentosum* |
| *P_multicystogenum* | AS2 | S.I. Kawikami | *Het_multicystogenum* |
| *P_patagonicum* | MYA-383 | J.C. Cavender | *P_patagonicum* |
| *P_pseudocandidum* | TNS-C-91 | H. Hagiwara | *Het_pseudocandidum* |

List of *Dictyostelium* species and strain identifiers using both the original taxon names of the species diagnosis and the taxon names of the recent re-classification (Sheikh et al., 2018). (*A_: Acytostelium; D_: Dictyostelium; P_:Polysphondylium; Cav_: Cavenderia; Hag: Hagiwaraea: Het_:Heterostelium; Rap_:Raperostelium).* The researchers who kindly sent us these species are indicated.

**Table A2. Oligonucleotide primers used in this work**

| Primer name | DNA sequence |
|---|---|
| Agl2-1501f-G4 | 5'-TGGATGGATAARATGGGTGA-3' |
| Agl2-1710r-G4 | 5'-CAAACCCAYTCWGGACCA-3' |

| | |
|---|---|
| AmdA-289f-G4 | 5'-GATACYCAYGTYCAYCAYTC-3' |
| AmdA-693r-GA | 5'-GCRATACTGTAYTCYTCCAT-3' |
| AmdA-693r-GB | 5'-GCRATAGAGTAYTCYTCCAT-3' |
| PurD-511f-G4 | 5'-GGATGTGCWGCAGGTAAAGGTG-3' |
| PurD-1321r-G4 | 5'-GTGATACCWCCACCDGTGATRTG-3' |
| PurL1-121f-G4 | 5'-CGTATGAATTTYACWACWACMTATTC-3' |
| PurL1-504r-G4 | 5'-WGARCCACCACCCATACCAAT-3' |
| PurL2-812f-G4 | 5'-AATTGGATGTGGGCTGCYAAA-3' |
| PurL2-1256r-G4 | 5'-GCCATYAATTGRCAACCATTRCA-3' |
| RpaA-779f-G4 | 5'-ATGATGGGTAARCGTGTYAATTAT-3' |
| RpaA-1114r-G4 | 5'-TCWARAATACCRGCMAMCATTTCATT-3' |
| SmdA1-299f-G4 | 5'-ATTTAATTGATCATCTAGATGAWTAT-3' |
| SmdA1-299f-Pvio | 5'-CTAGTCGATCATTATCAAGAGTA-3' |
| SmdA2-462r-G4 | 5'-CCATAAWCCRAAATAATTTGM-3' |
| SmdA2-462f-G4 | 5'-KCAAATTATTTYGGWTTATGG-3' |
| SmdA-666r-G4 | 5'-AAAGAGCCAATTTGATTTTAA-3' |
| Amd-443-F-g3/4 | 5'-AGAAAGATGARYGARTGGGA-3' |
| Amd-443-R-g3/4 | 5'-TCCCAYTCRYTCATCTTTCT-3' |
| Agl1-356-fw | 5'-TTGGTWTGGAAYCAYACWGC-3' |
| Agl1-648-rev | 5'-CATTTTNACRCAARCACCCCA-3' |
| Agl-648-R-G3 | 5'-MADTTTAACACAATCACCCCA-3' |
| Agl-1501-F-G3 | 5'-TGGATGGAYAAAATGGGWGA-3' |
| Agl-1710-R-G3 | 5'-CCAAACCCAYTCYGGACCA-3' |
| AmdA-289-F-G3 | 5'-GACACTCAYGTTCAYCATTC-3' |
| PurD-511-F-G3 | 5'-GGWKKTGCTGCTGGWAAAGGTG-3' |
| PurD-1321-R-G3 | 5'-GTRATACCACCACCWGTRATRTG-3' |
| PurL-121-F-G3 | 5'-CGTATGAATTTYACWACTACMTAYTC-3' |
| PurL-504-R-G3 | 5'-GCACTRCCACCWAACATACCAAT-3' |
| PurL-812-F-G3 | 5'-AACTGGATGTGGGCTGCYAAA-3' |
| PurL-1256-R-G3 | 5'-GCCATCAATTGACAACCATTACA-3' |
| Rpa-779-F-G3 | 5'-ATGATGGGTAAGAGAGTAAACTAT-3' |
| Rpa-1114-R-G3 | 5'-TCWARRATACCAGCNANCATTTCATT-3' |
| SmdA-299-F-G3 | 5'-GAYCTAATKGATCATTTRGAYGAAT-3' |
| SmdA-462-F-G3 | 5'-TCAAATTWCTTTGGNTTRTGGC-3' |
| SmdA-462-R-G3 | 5'-GCCAYAANCCAAAGWAATTTGA-3' |
| SmdA-666-R-G3 | 5'-AAATAACCAWRMTGATTTCAATTG-3' |
| Agl-356fw-G2 | 5'-TTGGTTTGGAATCAYACTTC-3' |
| Agl-356fw-G2a | 5'-CTCGTCTGGAAYCACACSGC-3' |
| Agl-356fw-G1 | 5'-CTYGTGTGGAAYCAYACTGC-3' |
| Agl-648re-G2 | 5'-ACACAATCKCCCCA-3' |
| Agl-648re-G1 | 5'-ACRCAGTCRCCCCA-3' |
| Agl-1501fw-G2 | 5'-TGGATGGACAARATGGGHGA-3' |
| Agl-1501fw-G1 | 5'-TGGATGGACAAGATGGGMGA-3' |
| Agl-1710re-G2 | 5'-CCAYARCCAYTCRGGTCC-3' |
| Agl-1710re-G1 | 5'-CCAKAKCCATTCKGGWCCG-3' |
| AmdA-289fw-G2 | 5'-GAYACTCACGTWCAYCAYTC-3' |
| AmdA-289fw-G1 | 5'-GATACYCACGTYCAYCAYTC-3' |
| AmdA-443re-G1/2A | 5'-TCCCAYTCGCTCATCTTTCTDCC-3' |
| AmdA-443re-G1/2B | 5'-TCCCAYTCGCTCATCTTGCGDCC-3' |

| | |
|---|---|
| AmdA-443fw-G1/2A | 5'-GGHAGAAAGATGAGCGARTGGGA-3' |
| AmdA-443fw-G1/2B | 5'-GGHCGCAAGATGAGCGARTGGGA-3' |
| PurD-511fw-G2 | 5'-GGATGYGCCGCYGGMAAAGGTG-3' |
| PurD-511fw-G1 | 5'-GGATGYGCYGCYGGWAAGGGTG-3' |
| PurD-1321re-G2 | 5'-GTGATACCRCCRCCRGTGATRTG-3' |
| PurD-1321re-G1 | 5'-GTGATACCWCCWCCRGTRATGTG-3' |
| PurL-121fw-G2 | 5'-AGAATGAAYTTCACMACYACCTACTC-3' |
| PurL-121fw-G1 | 5'-AGAATGAAYTTYACHACMACYTATTC-3' |
| PurL-504re-G2 | 5'-GCRGARCCRCCACCCATRCCGAT-3' |
| PurL-504re-G1 | 5'-GCRGMRCCACCACCCATACCRAT-3' |
| PurL-812fw-G2 | 5'-AACTGGATGTGGGCTGCCAA-3' |
| PurL-812fw-G1 | 5'-AAYTGGATGTGGGCWGCCAA-3' |
| PurL-1256re-G2 | 5'-GCCATCARYTGRCARCCGTTGCA-3' |
| PurL-1256re-G1 | 5'-GCCATCARYTGRCAACCRTTGCA-3' |
| Rpa-779fw-G1/2 | 5'-ATGATGGGYAAGCGTGTCAACTA-3' |
| Rpa-1114re-G2 | 5'-TCGAGRATRCCMRCNARCATYTCRTT-3' |
| Rpa-1114re-G1 | 5'-TCRAGGATACCTGCRASMAWYTCATT-3' |
| SmdA-299fw-G2 | 5'-GAYCTCGTYGAYCAYATCGAYGARTA-3' |
| SmdA-299fw-G1 | 5'-ATYTATGTGATCATATWGATGATTA-3' |
| SmdA-462re-G2A | 5'-TCCAMAGNCCAAAGTAYTTGGC-3' |
| SmdA-462re-G2 | 5'-TCCAMAGNCCAAAGTAYTTTGA-3' |
| SmdA-462re-G1 | 5'-TCCAYAARCCAAARTARTTGGC-3' |
| SmdA-462fw-G2A | 5'-GCCAATTACTTTGGNCTKTGGA-3' |
| SmdA-462fw-G2 | 5'-TCAAAYTACTTTGGWCTKTGGA-3' |
| SmdA-462fw-G1 | 5'-GCCAAYTAYTTTGGYTTRTGGA-3' |
| SmdA-666re-G2 | 5'-TCRAABAGCCARAGTGAYTT-3' |
| SmdA-666re-G1 | 5'-TCRAABARCCARAGTGAYTT-3' |

**References**


Kawakami, S., Hagiwara, H., 2008. Polysphondylium *multicystogenum* sp. nov., a new dictyostelid species from Sierra Leone, West Africa. Mycologia 100, 347-351.

Revell, L.J., 2012. phytools: an R package for phylogenetic comparative biology (and other things). Methods in Ecology and Evolution 3, 217-223.

Romeralo, M., Skiba, A., Gonzalez-Voyer, A., Schilde, C., Lawal, H., Kedziora, S., Cavender, J.C., Glockner, G., Urushihara, H., Schaap, P., 2013. Analysis of phenotypic evolution in Dictyostelia highlights developmental plasticity as a likely consequence of colonial multicellularity. Proc Biol Sci 280, 20130976.

Schilde, C., Skiba, A., Schaap, P., 2014. Evolutionary reconstruction of pattern formation in 98 Dictyostelium species reveals that cell-type specialization by lateral inhibition is a derived trait. EvoDevo 5, 34.

Sheikh, S., Thulin, M., Cavender, J.C., Escalante, R., Kawakami, S.I., Lado, C., Landolt, J.C., Nanjundiah, V., Queller, D.C., Strassmann, J.E., Spiegel, F.W., Stephenson, S.L., Vadell, E.M., Baldauf, S.L., 2018. A New Classification of the Dictyostelids. Protist 169, 1-28.
